# Surgeons, Hospitals, and Patients: The Road to Standardisation

**Published:** 1917-01-06

**Authors:** John G. Bowman

**Affiliations:** Director of the American College of Surgeons.


### SURGEONS, HOSPITALS, AND PATIENTS.

### The Road to Standardisation.

By JOHN G. BOWMAN, Director of the American College of Surgeons.

Mr. John G. Bowman gave the following address in Philadelphia at the American Hospital Association in September last:—

On behalf of the American College of Surgeons permit me to say, right at the start, that we have accepted your kind invitation to meet with you to-day with great seriousness. In many essentials the aims of the American Hospital Association and of the American College of Surgeons are one and the same. We hope that our interchange and discussion to-day will be of lasting importance. We have in common a vast complexity of good intentions, and to talk them over together now and then is wise. More than this, we hope that this occasion will mean that we, as two powerful groups, shall work together, and that, by good team-work, we shall make a larger measure of our good intentions come true.

In order now that we think straight across the fields wherein our common purposes lie, let me review briefly the expressed purposes both of your Association and of the College. By your constitution you emphasise your aims toward the "promotion of economy and efficiency in hospital management." You are concerned with hospital construction, with finances and economics, with national and State legislation, and with systems of accounting. You are concerned also with medical organisation and medical education, with the training of nurses, and with out-patient work. In the last analysis, all of this means that your energies go toward the proper care of sick people and also toward the prevention of disease.

The College of Surgeons, on the other hand, is an association of 3,500 surgeons of the United States and of Canada. It is an infant among medical societies. But I trust that I may say with due modesty that your success and ours are closely related, and that in the last analysis the beginning and the end of the College of Surgeons is the proper care of sick people, especially as they may be in need of surgical service, and also the prevention of disease. Our common meeting grounds are wide.

But more specifically what is the College? It is an association pledged to the most idealistic programme ever expressed in any profession. Its programme includes the elevation of standards of medicine, moral as well as intellectual. It is a guarantee of no indefinite sort to the public of honest and competent surgery. It is an association to encourage scientific advance in medicine and surgery. It is a national force to put an end to guesswork in diagnoses, to put an end to unnecessary operating, and to blot out the vicious practice known as the division of fees. Better probably than any group of men and women who gather themselves together in this country, you know the extent of these evils and of the unnecessary pain and death that trail in their wake.

Just a word here about fee-splitting. You know what it is, and you know that any doctor guilty of this practice is, under the most sacred relations which exist among men, both a liar and a thief. Milder terms are inadequate. More than any other one factor, this practice is responsible for the unnecessary operating and for operations on the part of incompetent surgeons. In the old-fashioned way now of a heart-to-heart talk, let us ask ourselves some questions. What headway are we actually making toward the better practice of medicine? Are we satisfied with our efforts? How often in the struggle does the blood rush to our finger-tips?

About a month ago, near Nashville, I visited the Hermitage, which is the old plantation of Andrew Jackson. My friend who was showing me about the estate told me that Andrew, at his death, left a slave by the name of Alfred. Alfred lived on many years about the place. One day he was asked if he "reckoned" that the General went to Heaven. "I dunno, suh," Alfred replied; "he did if he wanted to." This is the philosophy which stirs me more than any other. We can do things if we want to. We can bring ideals down out of Heaven if we want to, and make them live in any community or in any hospital. The difficult thing is to want to, and to put the want into incisive action.

There is a social law which operates unconsciously among us to the effect that as our organisation in society becomes more and more complex, our individual freedom of action decreases. We attain freedom under this process, but not of barbaric variety. It is freedom in conformity to law. And with these changes comes an increasing tendency that our ways become fixed. The fact is, we are born into a society whose customs we accept as a child accepts the atmosphere it breathes. Increasingly we look upon the man with suspicion who would change our ways, and especially is this true in hospital organisations where the routine from cellar to garret is sharply defined. In fact, hospital folk scarcely have their equal in this country for their conservatism. For example, less than two years ago there was a hospital superintendent in the south-west who went from room to room each morning with a little pot of burning tar on the end of a wire. He was fumigating the rooms. Physicians and surgeons protested that at this date there were better ways of fumigation. But the hospital superintendent had always fumigated in this fashion, and it was only with a pathetic wrench to his conscience that he finally gave up the established custom. In another hospital of approximately two hundred beds the leading surgeon recently explained to me that they had always got along very well without case-histories of any description. "Why," he asked, "should we disturb our success now by introducing this new stuff?" Again, from one coast to the other to-day many hospital trustees are sitting quietly by, allowing surgical operations to be performed in their respective hospitals after the merest guesswork in diagnosis. They defend themselves in this matter with the feeble statement that such procedure has always been the custom. Any hospital trustee or hospital superintendent to-day who sits quietly by and allows obviously incompetent work to go unquestioned or who tolerates the division of fees is unworthy of his trust.

I do not mean to infer that the motives in any instance cited are wrong. The point is that we are bound in our philosophy, in our life policies, in our conception of right or rights, and even in our religions, by the customs into which, without our consent, we were cast. Only by degrees and through genuine leadership and new economic demands do we change our established custom. In a more primitive society, when the established ways of the folk became intolerable, a revolution ensued. Then the ways were changed. In these more modern times we are less harsh and less emphatic. We steer clear of revolution; we use perhaps greater intelligence. Our progress is characterised by progressive development. My purpose now is to ask you to free yourselves for a little while at least from the bonds of custom and to ask you to agree with me, in so far as you will, upon a programme of activity for the better welfare of those whose care is entrusted to us. Our business is to know what we want to do, and then fearlessly to do it.

Since the early days of the organisation of the American College of Surgeons it has been evident to the Regents of the College that they must necessarily acquire accurate data with regard to the hospitals of this continent if they are to administer the College effectively and with fairness. Just a word further, now, in explanation of this matter. The College of Surgeons is an organisation to which are admitted surgeons who are honest and who have proved themselves qualified in surgery. But what does it mean to be qualified in surgery? It is a fair statement, I believe, to say that 80 per cent. of the knowledge used by the surgeon in his practice he acquires during his internship and his assistantship in a good hospital. An educational function has been forced upon the hospitals almost without their realisation. As an administrative necessity, the American College of Surgeons is interested to know what training the hospitals exact of the young doctors in their service during this period. A tentative outline of this problem has already been issued by the College and is in your hands. Beyond question there is benefit to be had, both by yourselves and by the College, if together we take up this subject and go to the bottom of it. This is part of the task to which we must address ourselves if we are ever individually to express in our work our true personal equations.

Many other subjects within our common fields of activity call for similar treatment. For example, what is the right procedure in the management of a hospital? This question calls for a specific answer as to the duties of a board of trustees, of a superintendent, and of a hospital staff. Again, in a hospital, let us say of one hundred beds in a small city, what are the laboratory facilities and the equipment requisite for the adequate care of patients? What is the responsibility of such a hospital to the entire local profession and to the community? Is the staff to be "open" or "closed"? These, among many others, are the perplexing problems upon which we should turn our clearest moments of thought, and which we should, by exhaustive study, solve. Our request is that we solve such problems together.

During the next three years the College has planned to spend at least $20,000 (£4,000) a year in the study of such problems as I have mentioned. The College takes up this work, asking the goodwill and co-operation of all concerned. It pledges itself to proceed in a kindly and sympathetic spirit, to be helpful always rather than critical. On the other hand the programme would be quite ineffective if it were not carried out in a sound and scholarly way. The first step is to obtain accurate data, and then to marshal these data fearlessly into a simple, comprehensible, and useful form. Such a series of studies, I am confident, will serve as a powerful lever for the advance of our purposes. It will serve as a bond by which each of us may accomplish greater ends than is possible single-handed.

On behalf of the Regents of the College I am authorised to ask the American Hospital Association to appoint a committee to co-operate with the American College of Surgeons in this proposed series of studies. The Regents ask that this committee advise with them from time to time as to the most useful fields of investigation, ways of procedure, and distribution of the published studies. The Carnegie Corporation has kindly agreed to contribute $30,000 (£6,000) toward this work.

There is one other matter which I beg with all kindness to mention. It is fundamental to the success of the entire programme. In many a community to-day the medical profession is engaged in undue rivalry and jealousies. Local groups of doctors are divided into little cliques, which indulge in unkind or bitter criticisms of one another. Such a condition, where it exists, defeats the public support to which the men are really entitled. It defeats the spirit of progress. Not infrequently officers of hospitals are drawn into this petty quarrelling. Now all of this we can stop if we want to stop it. A Southern evangelist used to thrill his audience by saying, "Quit your meanness. How are you going to quit it? Why, jest quit it." We need to be happy twenty-four hours a day, and in our hearts to understand that days of happiness are the days which make us wise. Do you ever consider how changed a place your hospital would be if everybody in it began work every morning having forgiven everybody everything? In closing, I venture to say that such a hospital would never have a serious financial problem to meet, and that the death-rate would go down where it belongs.

